# Trans–Holocene Bayesian chronology for tree and field crop use from El Gigante rockshelter, Honduras

**DOI:** 10.1371/journal.pone.0287195

**Published:** 2023-06-23

**Authors:** Douglas J. Kennett, Thomas K. Harper, Amber VanDerwarker, Heather B. Thakar, Alejandra Domic, Michael Blake, Bruce F. Benz, Richard J. George, Timothy E. Scheffler, Brendan J. Culleton, Logan Kistler, Kenneth G. Hirth

**Affiliations:** 1 Department of Anthropology, University of California, Santa Barbara, Santa Barbara, California, United States of America; 2 Department of Anthropology, The Pennsylvania State University, University Park, Pennsylvania, United States of America; 3 Department of Anthropology, The University of British Columbia, Vancouver, British Columbia, Canada; 4 Department of Biology, Texas Wesleyan University, Forth Worth, Texas, United States of America; 5 Department of Anthropology, Texas A & M University, College Station, Texas, United States of America; 6 Department of Anthropology, University of Hawaii at Hilo, Hilo, Hawaii, United States of America; 7 Institutes of Energy and the Environment, The Pennsylvania State University, University Park, Pennsylvania, United States of America; 8 Department of Anthropology, Smithsonian Institution, Washington, DC, United States of America; National Cheng Kung University, TAIWAN

## Abstract

El Gigante rockshelter in western Honduras provides a deeply stratified archaeological record of human–environment interaction spanning the entirety of the Holocene. Botanical materials are remarkably well preserved and include important tree (e.g., ciruela (*Spondias*), avocado (*Persea americana*)) and field (maize (*Zea mays*), beans (*Phaseolus*), and squash (*Cucurbita*)) crops. Here we provide a major update to the chronology of tree and field crop use evident in the sequence. We report 375 radiocarbon dates, a majority of which are for short-lived botanical macrofossils (e.g., maize cobs, avocado seeds, or rinds). Radiocarbon dates were used in combination with stratigraphic details to establish a Bayesian chronology for ~9,800 identified botanical samples spanning the last 11,000 years. We estimate that at least 16 discrete intervals of use occurred during this time, separated by gaps of ~100–2,000 years. The longest hiatus in rockshelter occupation was between ~6,400 and 4,400 years ago and the deposition of botanical remains peaked at ~2,000 calendar years before present (cal BP). Tree fruits and squash appeared early in the occupational sequence (~11,000 cal BP) with most other field crops appearing later in time (e.g., maize at ~4,400 cal BP; beans at ~2,200 cal BP). The early focus on tree fruits and squash is consistent with early coevolutionary partnering with humans as seed dispersers in the wake of megafaunal extinction in Mesoamerica. Tree crops predominated through much of the Holocene, and there was an overall shift to field crops after 4,000 cal BP that was largely driven by increased reliance on maize farming.

## Introduction

The global transition from hunting and gathering to increasingly complex forms of food production sometime after ~11,000 years ago was among the most consequential transformations in the cultural and environmental history of our planet. Agricultural economies ultimately emerged during the Holocene from at least 11 core areas of plant and animal domestication, four of which are located in the Americas (Mesoamerica, the Andes, the greater Amazon Basin and eastern North America) [1]. In the Americas, processes involved in the evolution of mutualistic relationships between indigenous people and potential cultigens, once spread by extinct megafauna (e.g., tree fruits, squashes, teosinte) [2–9], included domestication or partial domestication of key cultigens (e.g. [10–12]), dispersal of domesticates or partial domesticates through exchange networks (e.g. [13, 14]), displacement of foraging and horticultural peoples by expanding agricultural populations (e.g. [15]), and the assimilation of local people into expanding agricultural groups [16]. Major methodological advancements have occurred in recent years that enable researchers to examine the processes involved with the domestication, dispersal, and adoption of key cultigens. Microbotanical studies of pollen, phytoliths, and starch have revolutionized our ability to explore the earliest origins of key domesticates [14, 17, 18] or the changes in how they were processed (e.g., nixtamalization [19, 20]). Quantitative morphological studies of plant micro and macrobotanical remains have improved as the size of comparative collections has continued to increase [21] and ancient DNA studies have transformed our ability to document the domestication syndrome [1, 22–26]. The fact that mass spectrometry (AMS ^14^C) dating can accommodate smaller samples (50–100 mg rather than 10–20 g) [27] has played a major role in improving our understanding of the transition to food production by making it possible to date individual seeds. This has made it possible for researchers to construct more precise chronologies for domestication (e.g. [10, 28]). In this paper we provide a detailed chronological framework for these processes at the El Gigante rockshelter in Honduras.

El Gigante is one of a handful of dry rockshelters in Mesoamerica with well-preserved botanical materials whose dates span the transition from foraging to farming. These rockshelters provide a rare glimpse of early foraging strategies and changes in subsistence during this transition. Caves or rockshelters in Tehuacán (Coxcatlán, El Riego, San Marcos) [29, 30], Oaxaca (Guilá Naquitz) [31] and, peripheral to Mesoamerica, in Tamaulipas (Ocampo Caves) [32] exhibit regionally specific views of early foraging strategies and together provide valuable data about the transition to agriculture in Mesoamerica. Direct radiocarbon dating (^14^C) of macrobotanical remains from dry rockshelters supply important chronological control that helps us understand the processes involved in early plant domestication. Guilá Naquitz in the Valley of Oaxaca contains the earliest domesticated squash (*Cucurbita pepo*, ~10,000 cal BP) [10] and maize (*Zea mays* ssp. *mays*, ~6,300 cal. BP) [12]. These deposits have also become critical molecular “time capsules”. Ancient DNA from maize cobs in the Tehuacán Valley provides evidence of partial domestication by 5,600 cal BP [33, 34] and maize cobs dated to 4,400 cal BP from Tamaulipas display allelic frequencies that are typical of contemporary maize [22].

El Gigante rockshelter is unique because of its location along the southern periphery of Mesoamerica and because it is at a lower elevation than the dry caves of central Mexico. It thus provides a lower elevation southern neotropical counterpart to the Ocampo rockshelter complex and the more arid areas north of the periphery of Mesoamerica [32]. Both sites provide information about Early and Middle Holocene foraging strategies and have already played an important role in how we think about the dispersal of important domesticates (e.g., maize) to the south and the north [22, 28, 32]. Early stemmed projectile point technology is well documented in Early Holocene strata from El Gigante [35, 36] and the disappearance of these tools during the Middle Holocene mirrors other areas in the Maya lowlands [37] and parallels increases in ground stone tools and the diversification of animal and plant assemblages [38].

The well-preserved sequence of macrobotanical remains in El Gigante thus serves as an important archive for interactions and the flow of domesticated plants between Mesoamerica, Central America, and South America. Because El Gigante is located outside the natural range of ancestral teosinte (*Zea mays* ssp. *parviglumus* and ssp. *mexicana*), the absence of introgression with these wild species may have played a role in solidifying the domestication syndrome by at least 4,300 cal BP [28] that parallels increased consumption of maize in the region [24]. Maize genomes from El Gigante cobs dating between 2,300 and 1,900 cal BP indicate that admixed lineages from South America were introduced northward through Central America [24]. Tree crops (e.g., ciruela (*Spondias*), avocado (*Persea americana*)) are also present in large numbers [36] and detailed morphological and molecular work will ultimately provide critical data regarding the development of agroforestry and forest management. In this article we provide a major update to the chronology of the rockshelter based on a Bayesian stratigraphic assessment of 292 radiocarbon dates (a majority on identified macrobotanical remains) and provide a chronological framework for studying the tree and field crops present in the sequence. We start with a contextual overview of the rockshelter’s stratigraphy and excavation history.

### El Gigante rockshelter

**Fig 1:**
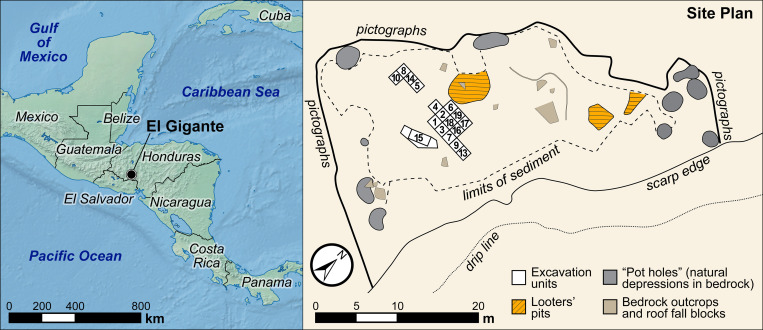
Map indicting the location of El Gigante rockshelter in western Honduras and planview map of the rockshelter showing the locations of test excavations and looters’ pits. All elements of the map come from Natural Earth (http://www.naturalearthdata.com/) and are compatible with the CC-BY 4.0 license.

El Gigante rockshelter is located in the highlands of western Honduras along the Estanzuela River (88.06°W, 14.22°N, 1300 masl) (Fig 1). River downcutting of the Miocene/Pliocene bedrock tuff formed the large rockshelter (42 m wide, 17 m deep, 12 m high), which is now protected from flooding on an elevated shelf. Dry conditions inside the dripline have resulted in well-preserved and relatively undisturbed archaeological deposits, which consist of ceramics, stone tools, animal bone, textile fragments and desiccated plant remains, including fragments of woven mats [36].

**Fig 2:**
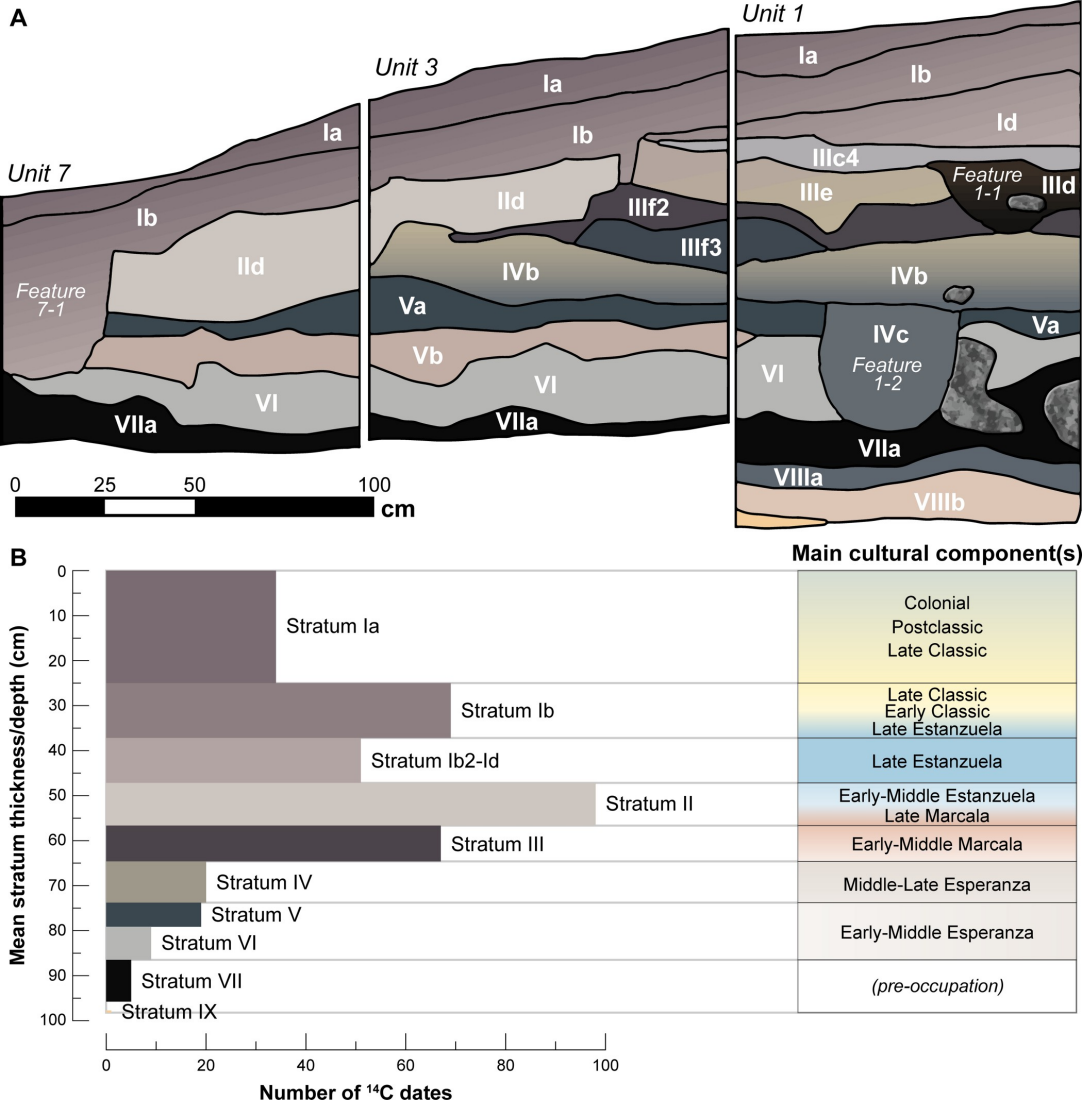
An example stratigraphic profile and associated distribution of radiocarbon dates. A. Intact stratigraphy at El Gigante, Units 1, 3 and 7 (south profiles; 2001 excavations). B. Stratigraphic distribution of ^14^C-dated materials, with excavated mean stratum thickness and superpositioning of main cultural components (data in S4 Table in S1 File).

Archaeological interest in El Gigante began in the early 1990s when looting activity was detected at the site. Formal investigations, led by George Hasemann and the Instituto Hondureño de Antropología e Historia (IHAH), commenced in 1993; Christine Hensley-Sherman and Anne Jung did the surface collection and test excavation that year [38]. Hasemann returned again in 1994 to excavate an adjacent unit. He uncovered more extensive evidence of the site’s stratigraphic record and, crucially, the presence of large numbers of preserved paleoethnobotanical remains. After Hasemann’s death in 1998, the largest excavations to date were carried out by Timothy Scheffler during the period June 2000 to March 2001 [36]. Scheffler excavated 18 new 1×1 m units at the southwestern end of the shelter (Fig 1) and revisited Hensley-Sherman and Hasemann’s prior excavations in Unit 15. These excavations revealed artifacts related to the Esperanza (Paleoindian), Marcala (Archaic) and Estanzuela (Formative, Early Classic) periods and several intrusive Classic period pits and burials. Stratified deposits at El Gigante extend as far as 2.5 m below the surface. Scheffler identified four non-cultural strata (VI–IX) that were identified at the base of the deposits overlain by five cultural deposits (I–V) (Fig 2A). Cultural strata range in thickness between ~5 and 25cm (Fig 2B). Cultural deposits were well stratified and contained a complex array of hearth and pit features. Looters’ pits dot the site, mainly in peripheral areas that may have contained mortuary and cache deposits [38]. In this earlier study, a handful of radiocarbon dates demonstrated that the site was occupied during multiple phases in the last 10,000 years. Our work builds upon Scheffler’s original chronological work described below [36, 38].

## Methods

### Radiocarbon sample from El Gigante

Scheffler presented 18 radiocarbon dates that included three dates that Hasemann obtained during his investigations of the site in 1994. Fourteen dates were on charcoal, 3 dates on macrobotanical remains, and one on a piece of plant fiber cordage [38]. Scheffler identified three phases in this early study: Esperanza (Paleoindian, 10,040–9,100 cal BP, Strata IV–V), Marcala (Archaic, 7,350–6,050 cal BP, Stratum III) and Estanzuela (Formative and Early Classic, 3,900–1,500 cal BP, Strata I–II). He inferred that the site was used very little outside of these episodes, and that occupation mostly ceased after the Early Classic [38]. However, this assessment was based on only a small number of ^14^C samples and was complicated by disturbances (both ancient and modern) and a general paucity of temporally diagnostic artifacts, especially in upper strata of the site. Since the late 2000s, the ^14^C corpus from El Gigante has grown by 357 dates and now totals 375. Direct dating of a large number of samples has made it possible to characterize numerous distinct use events and identify closer associations between these events and the site’s complex set of strata and sub-strata.

The existing dates currently consist of 49 from the Arizona Accelerator Mass Spectrometry Laboratory (AA) (Blake M, Benz B [unpublished]), 17 from Beta Analytic (Beta), 2 from the Illinois State Geological Survey (ISGS) [38], 187 from the Penn State Accelerator Mass Spectrometry Lab (PSUAMS), and 120 from the W.M. Keck Carbon Cycle Accelerator Mass Spectrometer Lab at UC Irvine (UCIAMS). We previously reported 108 of these dates [28], and an additional 218 were obtained between 2018 and 2021 with funding from the National Science Foundation and are reported here for the first time. We present all of the existing ^14^C data from El Gigante (S1 Table in S1 File), calibrated according to the IntCal 20 calibration curve [39] in OxCal v. 4.4 [40].

### AMS ^14^C dating

Macrobotanical samples directly dated by PSUAMS and UCIAMS (307 dates) were prepared for AMS ^14^C dating at the Human Paleoecology and Isotope Geochemistry Lab at the Pennsylvania State University using published sample purification protocols [27]. Samples were cleaned and pretreated according to the standard acid/base/acid (ABA) process involving 30-minute baths in 1N HCl and NaOH at 70°C followed by a final acid wash to remove carbonates that can form during the process. Samples were then returned to neutral pH with two 15-minute baths in ultrapure water at 70°C to remove chlorides, then dried on a heater block. They were then combusted at 900°C for 3 hours in evacuated sealed quartz tubes using a CuO oxygen source and Ag wire to remove chloride compounds. Primary (OX-2) and secondary (FIRI-D/F, FIRI-H) standards and a Queets Wood background were selected to match the sample age and underwent the same chemical steps for quality assurance. Graphitization of CO_2_ was carried out using a modified hydrogen reduction method [41]. AMS ^14^C measurements were conducted at the PSUAMS and UCIAMS facilities. All ^14^C ages were δ^13^C-corrected for mass-dependent fractionation with measured ^13^C/^12^C values [42]. The AA, Beta, and ISGS labs used comparable sample purification techniques (see laboratory websites for graphitization and measurement protocols).

### Chronological analysis and Bayesian sequencing

Cave and rockshelter sites are famous for their oftentimes difficult and discontinuous age-depth profiles. This issue is compounded by shallow stratigraphic layers and redeposition of materials due to frequent site use and re-use. The prevalence of disturbance at El Gigante led us to adopt a hybrid approach to understanding the site’s chronology. We considered issues of stratigraphic integrity on the one hand while also dealing with directly dated materials in a manner independent from stratigraphic considerations. We considered two sub-sets of data: 369 dates that constitute the total ^14^C sample (excluding six modern and pre-habitation outliers) and 292 dates that constitute the sample that originated from intact strata that exhibit proper superpositioning. We use the larger set of dates to characterize the depositional history of the entire site as well as different categories of directly dated archaeobotanical remains. The latter, smaller set of dates forms the basis for a Bayesian chronological model of the site’s stratigraphic development, which we use to infer the age of 9,429 paleoethnobotanical specimens that are not directly radiocarbon dated.

Previous Bayesian sequencing of the site using 88 dates expanded on the results of prior studies and produced a model that exhibited substantial Paleoindian, Archaic, Formative, and Classic period occupations [28]. Expansion of this subset to 292 dates adds considerable detail and chronological resolution, bringing the site’s sequence to 18 modeled phases (S2 and S3 Tables in S1 File). Several of these phases, especially in the mixed layers corresponding to the most intensive period of rockshelter use, are not readily discernible in the stratigraphic record.

**Fig 3:**
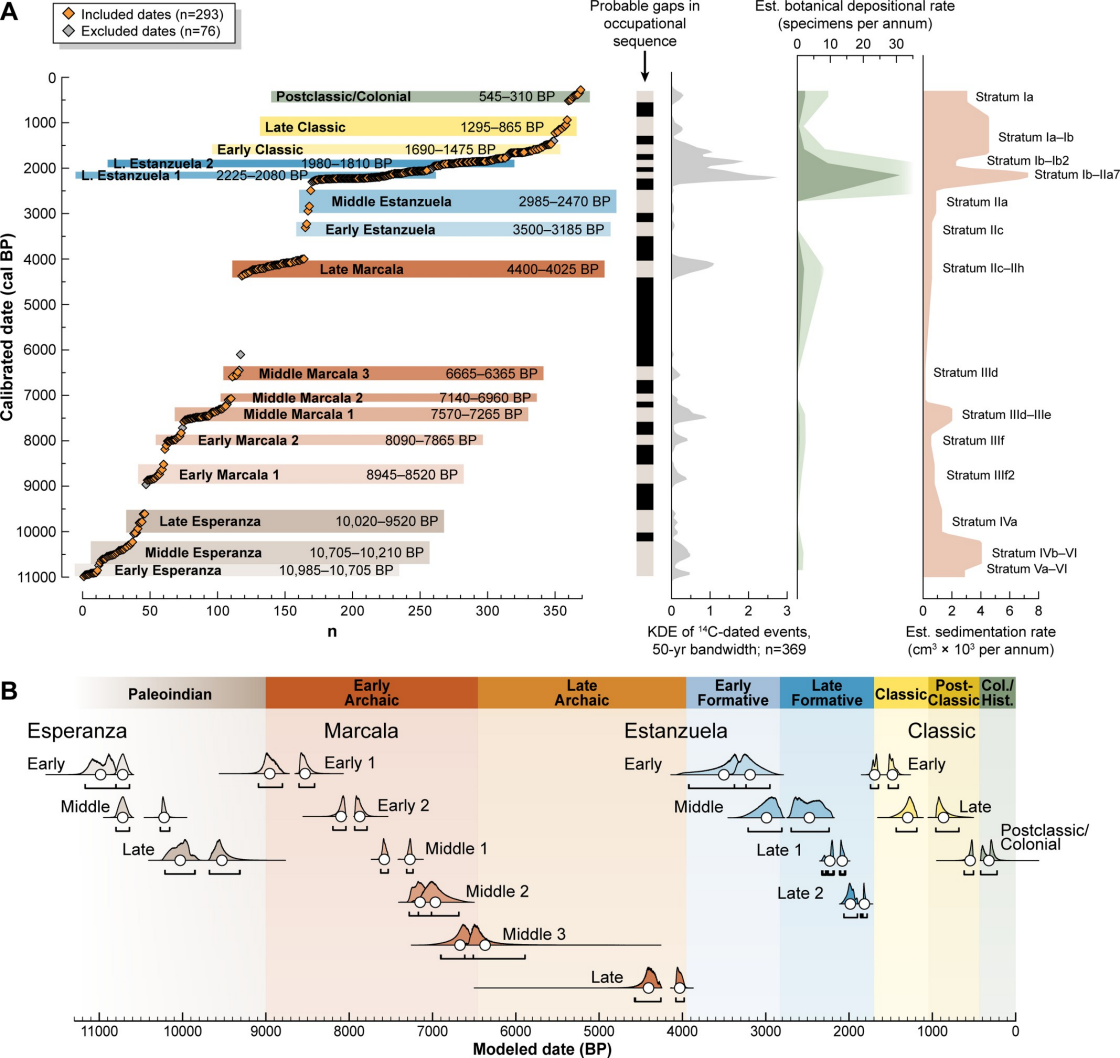
The distribution of all 369 ^14^C dates from El Gigante rockshelter. A. Calibrated dates are grouped according to their inclusion or exclusion in the chronological model and categorized by model phase. The relative depositional intensity of these habitational episodes is generated using kernel density estimation (KDE; light gray) of calibrated ^14^C dates with a 50-year bandwidth. This record compares favorably with additional proxy records: the depositional rate of paleobotanical specimens (green with light green shading showing 4x exaggeration so earlier trends are visible) and the rate of sedimentation across all units (light orange). B. Sequenced radiocarbon chronology displaying modeled occupational phases shown relative to the regional cultural chronology. Probability distributions for all ^14^C dates and the modeled phase boundaries are provided in **S1 Fig OxCal model, graphical output**. Given the extensive nature of the sampled materials, gaps present in the ^14^C record are likely indicative of gaps in the site’s occupational sequence. The raw data, model output and model code can be found in S1–S3 Tables in S1 File.

The stratigraphic concordance derived from Scheffler’s excavations [38] provided an excellent set of *a priori* assumptions on which to build a model. From this starting point, the specification of model phases was an iterative process performed in reference to the agreement (A) and convergence (C) indices in OxCal 4.4, which are sensitive to the temporal spacing between observations in a phase (i.e. depositional intensity). Model boundaries were defined according to the temporal continuity or discontinuity between habitational episodes, with continuous use defined by a single boundary (representing an inflection point in use intensity) and discontinuous use defined by a double boundary (representing a break in site use). Phase boundaries are probabilistic in nature and vary in accuracy according to the number and precision of the constituent dates (summarized in Fig 3B and S2 Table in S1 File). In order to generate discrete phases for analysis of the paleoethnobotanical sample we define model phases as being the duration between the mean value of the start and end modeled boundaries for each phase.

We omitted 82 dates from the model. The largest number (68) were excluded because of looting or stratigraphic disturbance, mostly the result of extensive re-modeling of the rockshelter’s floor and re-deposition of materials that occurred during the Late Formative period. If substantial ambiguities in stratum assignments were present and documentary evidence did not support assignment of dates to a more chronologically “appropriate” adjacent stratum, dates were omitted. Twenty-five excluded dates originated from Units 5 and 7 alone; while intact strata exist in some sections of these units, large portions were significantly disturbed by modern looting. We omitted four dates because they predated the occupation or were modern outliers and three dates because they were missing provenience information. We removed a further seven dates from the sequence because of poor model agreement (using the manual outlier detection method described in [43]). The number of ^14^C dates in cultural strata range between ~20 (Stratum V) and 100 (Stratum II; Fig 2B).

### Proxy records for site and crop utilization

The nature of the ^14^C sample from El Gigante, which prioritizes direct dating of paleobotanical materials (especially maize), raises questions about whether results are representative of the overall use-life of the site. In order to examine whether the ^14^C sample is representative of the site’s habitational record, we generated time series that describe the density of ^14^C-dated events and rates of botanical deposition and sedimentation. Our assumption was that all three of these proxy datasets should be correlated.

We summarized radiocarbon-dated events (calibrated, but not modeled) with a kernel density estimation (KDE) that has bandwidth of 50 years, calculated according to the standard approach:

$$f(x)=\frac{1}{nh}\sum_{i=1}^{n}K\left(\frac{x-x_{i}}{h}\right)$$ (Eq 1)

where *h* is the bandwidth, *n* is the sample size, *K* is the standard normal distribution function, *x* is the time reference, and *x*_*i*_ is the radiocarbon age, defined as each date’s mean intercept with the calibration curve. The intent behind using a KDE approach in this case is to render each ^14^C-dated sample as a uniformly distributed event, thus alleviating concerns over differential uncertainty and calibration curve effects in the use of ^14^C probability distributions as a proxy for site use, which can exaggerate high-precision data and adopt multi-modal distributions in response to calibration curve reversals.

The estimated sedimentation rate was determined by measuring stratum thicknesses at the corner of each of Scheffler’s (2008) unit profiles (S4 and S5 Tables in S1 File) [38]. An average of each of these measurements then provides an interpolated value for stratum thickness across each 1×1 m unit. The sedimentation rate for a given stratum or sub-stratum (*s*) can be modeled as where *z* is stratum thickness in a given unit *n* and *d* is the total

$$s=\left(\frac{\sum_{i=1}^{n}z_{i}}{d}\right)$$ (Eq 2)

minimum bounding span of ^14^C-dated events originating within the stratum. Site-wide sedimentation rate at a given time reference *t* (*S*_*t*_) is determined by summing all values of *s* where *t* intersects with the temporal span of *d*. This accommodates for chronological overlap between many designated strata and sub-strata.

Paleoethnobotanical specimens were assigned to chronological phases based on consideration of their individual excavation contexts, including unit, level and stratum designations (S6 Table in S1 File). The estimated botanical depositional rate was calculated according to a summation model almost identical to that described for the sedimentation rate, with the following exceptions: 1) phases and temporal durations are taken from the sequenced site chronology; and 2) a lack of chronological overlap means that we can omit summing the rates of all of the corresponding strata for a given time reference. We calculated an index of tree crops to field crops, relevant to discussion of the relative importance of different cultivars across the use-life of El Gigante. The index was calculated according to the ratio of these observations and scaled from -1 (indicative of 100% tree crops) to +1 (indicative of 100% field crops).

We resampled the KDE and calculated sedimentation and depositional rates at 50-year intervals using the PAST software package, v. 4.09 [44]. This resulted in time series with n = 215 observations that spanned 11,000–300 years (S7 Table in S1 File).

## Results

The extremely high resolution and density of ^14^C-dated events at El Gigante enables us to discern small perturbations in the distribution of dates that may indicate new occupational phases and sub-phases (Fig 3). Some of the 18 phases modeled in OxCal are discrete while others are continuous. There are also major gaps in the record when the rockshelter was not in use. The model boundaries are representative of inflection points in the distribution of likely events when the rockshelter was in use. Our model includes sub-phases delineated according to variations in depositional intensity during the Late Marcala (Late Archaic Period) and Late Estanzuela 2 (Late Formative Period) phases (dubbed Late Marcala (a)) and (b) and Late Estanzuela 2(a) and 2(b), S2 Table in S1 File), but they are combined here for the purposes of presenting the overall site chronology. Calibration curve reversals that are present at ~2,300 and 1,700 cal BP (time references representing transitions to the Late Formative and Early Classic occupations, respectively) likely affected phase boundaries during the most intensive period of site use, making it difficult to discern the accuracy of modeled gaps during this interval. Convergence indices dip to ~97% in these regions but remain above the 95% acceptance threshold. Overall model results exhibit good agreement and convergence indices between the data and inputted parameters (A_model_: 85.7; A_overall_: 105.5).

The KDE of ^14^C-dated events corroborates the observation of approximately 16 occupational phases. This record exhibits a moderate-to-strong correlation with estimated sedimentation (*r* = 0.544, *r*^2^ = 0.296, *p =* <0.001) and botanical deposition rates (*r* = 0.693, *r*^*2*^ = 0.481, *p =* <0.001) throughout the site’s use-life (S8 Table in S1 File). While care should be taken in assigning relative “intensity” of site use based solely on the density of ^14^C-dated events, the KDE complements our modeled sequence and highlights episodes of use that are difficult to discern in the stratigraphic record. Additionally, the divergence between KDE and sedimentation rates during the last ~2,000 years illustrates the extensive site disturbance and redeposition that occurred from the Late Formative to the present, which has had deleterious effects on preservation of many earlier, Archaic strata.

Paleoindian use of the rockshelter first occured during the Early and Middle Esperanza phases (10,985–10,705 modeled BP [henceforth BP] and 10,705–10,210 BP, respectively; Strata VI–IVb). These phases account for 12% of the total dates and show a moderately dense distribution. After a gap of ~190 years, the rockshelter was used during the Late Esperanza phase from 10,020–9,520 BP (Stratum IV and Strata IV/III interface). The density of observations decreases here, indicating that use of the site by Paleoindian groups was reduced, and then the site was abandoned for ~575 years. Esperanza levels are distinct because of the presence of stemmed bifaces with expanding and single side fluting that disappeared during the subsequent Archaic period Marcala phases [35, 36]. Paleobotanical remains are abundant in the radiocarbon sample from the beginnings of human use of the rockshelter, including squash, maguey and avocado.

Because of the paucity of diagnostic pottery throughout the Archaic and Formative period deposits at El Gigante, these phases of use are delineated according to lithic assemblages, by their positioning within the general Mesoamerican chronology, and, in later periods, by the presence of diagnostic macrofossils such as maize. Archaic period Marcala phase use of the site was highly episodic. The first episode of use, designated Early Marcala 1 (Stratum IIIf2), lasted from 8,945 to 8,520 BP. After a gap of 430 years, the site was used from 8,090 to 7,865 BP, dubbed the Early Marcala 2 occupation (Stratum IIIf). The density of ^14^C-dated events suggests that the Early Archaic inhabitants used the site as intensively as it was used during the Paleoindian period. Use of the site intensified briefly during the Middle Marcala 1 phase (7,565–7,265 BP; Stratum IIIe–IIId), which provided more materials for dating than the previous two phases combined. Two ephemeral Middle Marcala phases are indicated by Feature 19-5b, a later pit dug into Early Marcala Stratum IIIf2 (dubbed Middle Marcala 2), and by a smattering of materials at the Stratum IIId/IId interface (Middle Marcala 3). These phases are dated to 7,140–6,960 and 6,665–6,365 BP, respectively. Following the end of Middle Marcala 3, there is a gap of roughly 1,955 years before the rockshelter was used again, which dates to the Late Marcala phase of the Archaic Period and mostly associated with Strata IIh–IIc. The Late Marcala ^14^C record (4,400–4,025 BP) suggests intensive deposition over a relatively short period of time. This observation is inconsistent with results from the sedimentation model, which are biased by Formative-period disturbance and redeposition of sediment and materials. The Late Marcala macrofossils include an abundance of maize cobs, which account for half of all of the Late Marcala ^14^C-dated materials.

The Early and Middle Formative (Estanzuela) phases at El Gigante are characterized by ephemeral use of the site and are dated by two charcoal lenses from Units 6 and 18. These phases are tentatively dated to 3,500–3,185 and 2,985–2,475 BP, respectively, and are most likely associated with the upper reaches of Stratum II (IIc–IIa). In the Late Formative Period the Late Estanzuela is divided into two sub-phases, Late Estanzuela 1 (Stratum IIa7–Ib; 2,225–2,080 BP) and Late Estanzuela 2 (Stratum Ib2–Ib; 1,980–1,810 BP). From Late Estanzuela 2 onward, all events at the site pertain to Stratum I, a thick, mixed stratum. The 115 Late Formative dates, of which 87 come from maize cobs, account for 39% of the total sample of dates used in the model. In places, activities at the site substantially disrupted earlier Archaic period strata; numerous Marcala phase outliers are present in Stratum I deposits that date to the Late Formative and Early Classic. The high density of ^14^C-dated events and high rate of sediment deposition/redeposition observed at El Gigante strongly suggest that the site’s most intensive use occurred during the Late Estanzuela period. One notable intrusive feature that dates to this period is a human burial in Unit 10 that, based on a sample of painted textile, dates this burial to the beginning of the Late Estanzuela 1 phase (2,345–2,155 cal BP, 2σ, UCIAMS-108395). Unfortunately, this burial was destroyed by modern looting.

The density of ^14^C-dated events during the Early Classic occupation of the site (1,690–1,475 BP) is substantially lower than during the Late Estanzuela peak. While maize continued to be a main component of the paleoethnobotanical assemblage, remains that date to the Early Classic include a higher proportion of avocados and wild fruits. After a gap of ~165 years, a few deposits date to the Late Classic (1,295–865 BP), represented by six AMS ^14^C dates. These samples consist of maize cobs scattered across Units 1 and 19 and an intact pit feature containing a deer-hide bag and an avocado seed. After the end of the Late Classic occupation, there is a 315-year gap in occupation.

Postclassic and Colonial-period use of the site was likely minimal. These phases (545–310 BP) are almost entirely represented by acorns recovered from solution pits (water-worn holes in the bedrock along the drip line of the rockshelter). However, one Postclassic maize cob and two Early Colonial gourd rinds attest to ephemeral human use of the rockshelter during this time.

## Discussion

**Fig 4:**
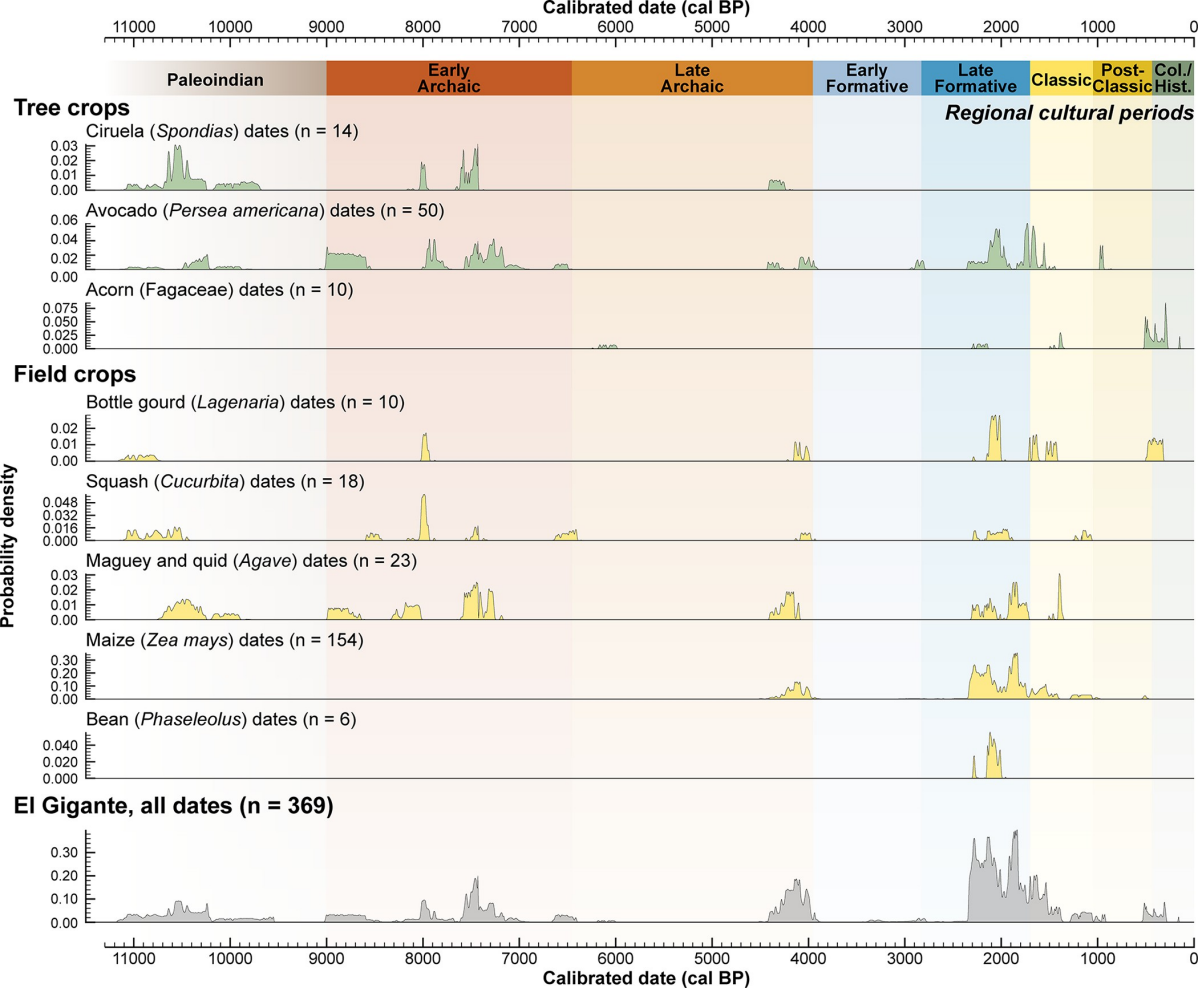
Summed probability distributions of dated paleobotanicals from El Gigante rockshelter. Genera representative of tree crops, field crops, and other agricultural domesticates are compared against the regional archaeological chronology and the summed probability distribution of all the available radiocarbon dates for the rockshelter. Regional cultural periods defined in Fig 3 are shaded to provide chronological context for the appearance of economically important plant species.

**Fig 5:**
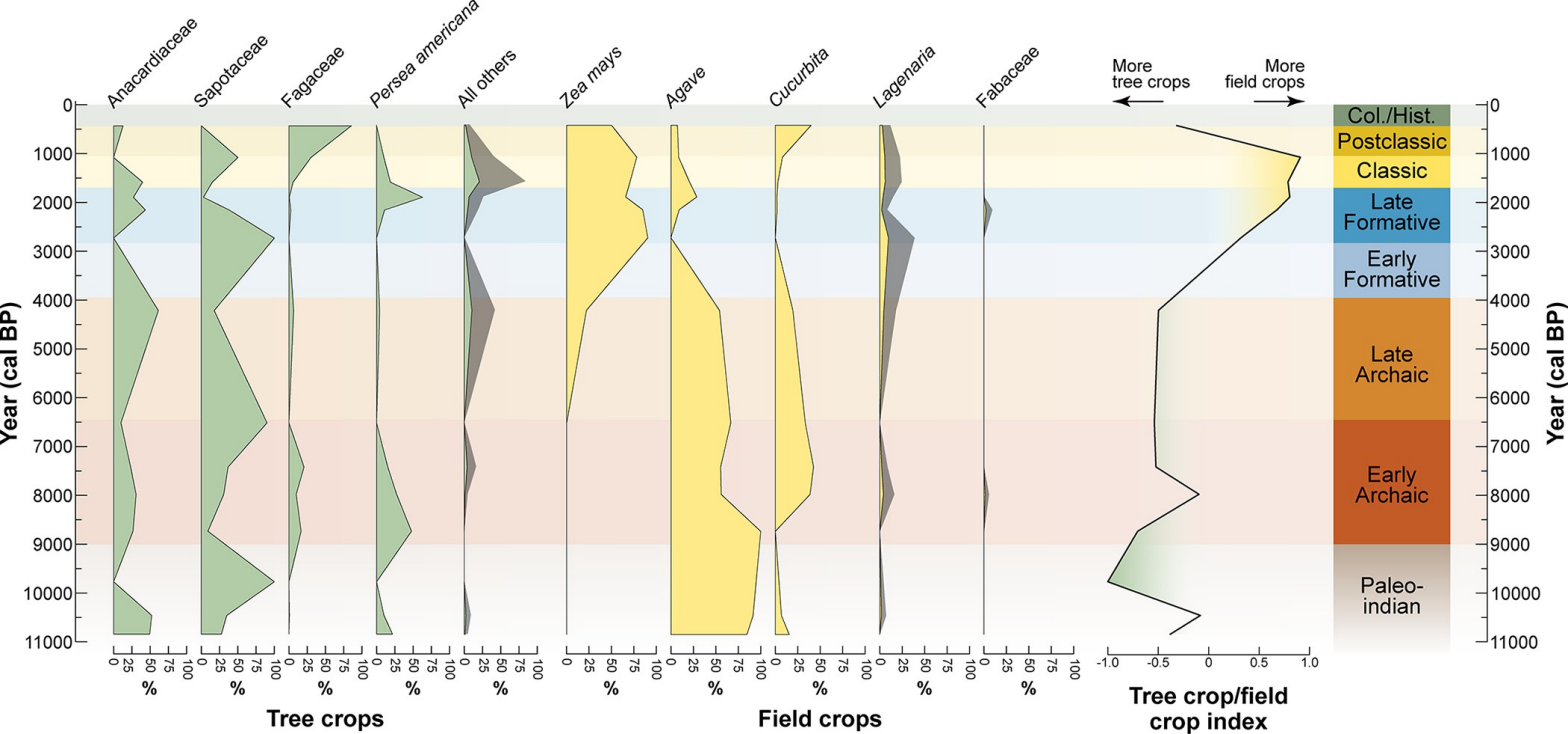
Percentages of select tree crops (green) and field crops (yellow) from the El Gigante rockshelter and an index of tree crop to field crop (TC/FC) use. Taxa with comparatively low counts are additionally plotted with a 4x exaggeration (gray) to show trends. Changing percentages of tree and field crops are shown relative to the regional archaeological chronology. This general index shows the use of field crops peaked during the Late Formative and Classic periods at El Gigante, while tree crops were predominant during all other periods.

El Gigante’s trans–Holocene deposits provide insights into the coevolutionary relationships involved in the domestication, adoption, and use of tree and field crops [24, 28, 36, 38]. In this article, we provide a more detailed Bayesian chronological framework for studying the full range of tree and field crops in order to explore the interrelationships between them over the last 11,000 years. Building this chronology involved directly radiocarbon dating large numbers of cultigens: bottle gourds (*Lagenaria* spp.), squashes (*Cucurbita* spp.), avocados (*Persea* spp.), hog plum (*Spondias* spp.), agave (*Agave* spp.), maize (*Zea mays*), and beans (*Phaseolus vulgaris* and *Phaseolus dumosus*) (Fig 4). In addition, our chronological model provides a framework for assigning date ranges to undated materials from the best preserved rockshelter contexts (Fig 5). We briefly discuss the significance of our chronological findings and will expand upon these initial observations with future morphological and genetic studies.

The El Gigante plant assemblage indicates that early forager-farmers in the region experimented with a range of potential tree crops throughout the Holocene (Fig 5). Sapotaceae (5 species, with *Pouteria* spp. dominating, 93%), Anacardiaceae (*Spondias* spp. or hog plum), and *Persea americana* (avocado) all have edible fruits and occur in the earliest Paleoindian cultural levels (~11,000 cal BP). They also persist through the Holocene. Small numbers of other tree species include *Annona cherimola*, *Annona squamosa*, *Annona muricata*, *Rollinia mucosa*, Brahea sp., *Bactris* sp., *Acrocomia* sp., *Attalea* sp., cf. *Copaifera* sp., cf. *Reinhardtia* sp., *Talisia* sp., and *Celtis reticulata* and occur in low frequencies starting as early as the Paleoindian period (Fig 5). Both avocados and hog plums became important Mesoamerican tree crops [45, 46]. The earliest directly radiocarbon dated avocado remains in the Paleoindian period are 11,105–10,700 cal BP (2σ, PSUAMS-9358) in age and the earliest hog plum dates to 11,085–10,705 cal BP (2σ, UCIAMS-90929). Hog plum grows in the vicinity of the cave today and bears fruit during wet season months (September–October). Its presence persists throughout the Holocene (included in Anacardiaceae, Fig 5). Avocados also are present throughout the sequence and point to the economic importance of this tree crop through time.

The earliest acorns in the assemblage date to 6,200–6,000 cal BP (2σ, PSUAMS-8007). Acorn exploitation at El Gigante is not surprising given the surrounding pine-oak forest, even though significant labor investment was required to remove bitter-tasting tannins before consumption. Acorns recovered from Postclassic period solution pits could provide evidence for how they were possibly stored late in time [38]. Overall, the dominance of tree crops in the El Gigante sequence persisted until the end of the Archaic period ~4,000 BP (Late Marcala phase), when there was a shift to field crops during the Early Formative period that was largely driven by the increased importance of *Zea mays* during the Late Formative period (Late Estanzeula phase, Figs 3 and 5). The early and persistent importance of tree crops at El Gigante is consistent with incipient tree crop cultivation found in South America by 11,500 cal BP [18, 47, 48] and with the hypothesis that early Native American populations were the primary seed dispersers of these potential cultigens in the wake of megafaunal extinction [5].

Bottle gourds and squashes are the first cultivars or potential field crops to appear in El Gigante deposits during the Paleoindian period (~11,000 cal BP) and they persisted throughout the Esperanza (Paleoindian), Marcala (Archaic), Estanzuela (Formative) and Classic period occupations. Genetic work indicates that bottle gourds in the Americas originated in Africa [3]. They likely drifted across on ocean currents during the Pleistocene and were dispersed widely throughout the Americas, likely by megaherbivores during the Pleistocene. Our work at El Gigante now provides the earliest directly AMS ^14^C-dated *Lagenaria* bottle gourd remains in the Americas; dating between 11,150–10,765 cal BP (2σ, PSUAMS-8058). Early *Lagenaria* specimens in the Americas have also been identified at Guilá Naquitz in Oaxaca, Mexico and Little Salt Spring in Sarasota, Florida (USA) [3]. The plant was highly valued as a light weight container, and its importance in the vicinity of El Gigante persisted well after the introduction of ceramic technology ~3,600 cal BP during the Early Formative period [49]. Determining the domestication status of these early bottle gourds is complicated by the absence of extant wild *L*. *siceraria* for morphological and genetic comparison, but ancient DNA analysis of these specimens could help place them in the context of later cultivated gourds. Our results from El Gigante are consistent with this early potential cultigen entering into symbiotic relationships with early human populations in the region, and that they became the main agent of dispersal with megafaunal extinction [50, 51].

Our work has also produced the earliest directly radiocarbon dated macrobotanical remains of *Cucurbita* squash known in the Americas during the Paleoindian period (11,090–10,720 cal BP, 2σ, PSUAMS-8055). Squash distribution in the Americas was shaped by coevolutionary relationships with megaherbivores that were disrupted by Late Pleistocene animal extinctions [52]. Mutualistic relationships with early human populations resulted in a minimum of five domesticated species [53]. The earliest known domesticated squash (*C*. *pepo*) remains, which come from Guilá Naquitz, are directly radiocarbon dated to 10,035–9,905 cal BP (2σ; Beta-100766) [10]. Phytolith data suggests the domestication of multiple species soon after this time in Mesoamerica, Central America and South America (*C*. *moschata*, *C*. *ecuadorensis*) [14]. *Cucurbita* sp. microbotanical remains have been identified in Amazon lowland forest as early as 12,000 cal BP [18, 54], but domestication status is unclear in these earliest deposits. Microbotanical evidence for *C*. *ecuadorensis* from southern Ecuador and *C*. *moschata* in the Zaa Valley of Peru both date to ~10,000 cal BP [14]. Morphological work underway will determine the domestication status of the early squash remains from El Gigante, but these early dates demonstrate that the coevolutionary relationship with humans started early and is consistent with an interpretation that humans were early dispersal agents in the wake of megafaunal extinctions. *Cucurbita* squash remained the most important cultivar in the vicinity of El Gigante until the end of the Archaic Period (Late Marcala; ~4,000 BP), when maize cultivation started to eclipse its importance.

Agave (Agave spp.) is another crop that is evident throughout the El Gigante deposits starting in the Paleoindian period. By the Classic period (~1,400 BP) this crop was being cultivated in fields adjacent to houses at the site of Cerén in El Salvador [55]. The earliest AMS ^14^C-dated agave at El Gigante is a quid (a chewed fleshy and fibrous leaf) that dates between 10,655–10,300 cal BP (2σ, PSUAMS-5391). Evidence of this plant throughout the Esperanza (Paleoindian), Marcala (Archaic), Estanzuela (Formative), and Classic period deposits demonstrates that this plant was important to the economies of numerous communities over a long period of time. Thirteen directly ^14^C-dated quids confirm the persistent importance of this plant as a food source. Agave can be roasted or boiled into a soup and was also an important source of alcohol, fiber and building material historically [56, 57]. Early agave remains (~10,000 cal BP) are known from Tehuacán [58] and Guilá Naquitz in the Valley of Oaxaca [59]. Its distribution at these sites suggests that it was transplanted and possibly managed [58]. It remains unclear which species were domesticated and how these coevolutionary relationships developed. In the Maya region, henequin (*Agave fourcroydes* Lem.) was domesticated for its fiber [56]. The early and persistent use of agave registered at El Gigante points to a long-term coevolutionary relationship that warrants an ancient DNA study to unravel the history of this important cultigen.

Maize and beans are two important Mesoamerican field crops that occur in the El Gigante record after 4,500 cal BP (*Zea mays*, 4,525–4155 cal BP (2σ, AA-93157); *Phaseolus vulgaris*, 2,300–2,005 cal BP (2σ, PSUAMS-8042)). The addition of these completed the Mesoamerican triumvirate—maize, beans and squash, which were grown together for mutual benefit. Over 10,000 carbonized and uncarbonized maize macrofossils (e.g., cobs, leaves, stalks) have been recovered from these deposits. The bulk of these remains occur in Late Formative (Late Estanzuela phase) and Early Classic contexts (2,225–1,475 BP), but Late Archaic (Late Marcala phase; 4,400–4,025 BP) deposits contain a small number of cobs. We have directly dated 30 Late Marcala cobs and 120 post-Marcala cobs. The late Marcala cobs have 10–14 rows and overlap in size and row number with later cobs [28]. We have argued elsewhere that this suggests domesticated land races productive enough to be a staple grain were present by the Late Archaic period (~4,400 BP). This observation is consistent with dietary stable isotopes (δ^13^C and δ^15^N) that indicate increasing maize consumption from elsewhere in the region [60]. Ancient DNA from cobs dating to the Late Estanzuela phase (Late Formative period, 2,300–1,790 BP) indicate the backflow of admixed maize lineages introduced from South America [24]. The influx of germplasm, likely with increased admixture from the highland *Zea mays* spp. *mexicana* species [61], may have contributed to the development of a more productive staple grain and resulted in a dramatic shift in the importance of maize in the local subsistence economy after ~4,000 BP (Fig 5).

Beans are most abundant in El Gigante deposits during the Late Estanzuela 1 phase of the Late Formative period (2,225–2,080 BP) and persist through the Late Classic Period (1,295–865 BP). Wild beans (*Phaseolus* ssp.) were widely distributed through Central and South America [14, 54] and were domesticated multiple times during the Holocene [62]. Starch grains from northwest South America indicate bean cultivation by 9,600 cal BP [47, 63] and Lima beans (*Phaseolus lunatus*) and common beans (*Phaseolus vulgaris*) from the Andes have been directly radiocarbon dated to 5,600 cal BP and 4,400 cal BP, respectively [64]. In Mesoamerica, wild runner bean (*Phaseolus* spp.) exploitation is known from Guilá Naquitz (Oaxaca) dating as early as 10,600 cal BP [31]. Common beans (*Phaseolus vulgaris*) from Coxcatlán Cave (Tehuacán) date between 2,390–2,210 cal BP, which is roughly contemporary with the earliest common beans from Oaxaca (2,200–2,000 cal BP, [64]). Tepary beans (*P*. *acutifolius*) and butter beans (*P*. *coccineus*) are also present in Mesoamerica after this time. Genetic data point to a single domestication event for common beans in west-central Mexico [62]. DNA evidence also suggests the domestication of a single Mesoamerican lima bean (*Phaseolus lunatus* L.) in central western Mexico [65]. Future morphological and genetic work on the El Gigante beans will determine how they fit into the overall domestication process.

## Conclusions

The El Gigante rockshelter provides a remarkably well-preserved and extensive macrobotanical assemblage that enables researchers to examine the long term evolutionary and demographic processes involved with the domestication of multiple tree and field crops. Our work at El Gigante results in the following primary findings:

1. We use 375 radiocarbon dates, a majority of which are for short-lived botanical macrofossils (e.g., maize cobs, avocado seeds, or rinds) to define at least 16 discrete episodes of rockshelter use during the last 11,000 years, separated by gaps of ~100–2,000 years. Radiocarbon dates were used in combination with stratigraphic details to establish a Bayesian chronology for ~9,800 identified botanical samples spanning this interval.
2. We find evidence for the early and persistent importance of tree crops at El Gigante starting in the Paleoindian period at ~11,000 years ago. Sapotaceae (*Pouteria* spp.), Anacardiaceae (*Spondias* spp.), and *Persea americana* (avocado) were the dominant tree crops. The early importance of tree crops is consistent with the hypothesis that early Native American populations were the primary seed dispersers of these potential cultigens in the wake of megafaunal extinction.
3. We provide the earliest macrobotanical evidence for *Cucurbita* squash and bottle gourds (*Lagenaria*) in the Americas. These observations are also consistent with these cultigens entering into an early symbiotic relationship with humans that ultimately became the main agent of dispersal in the wake of megafaunal extinction.
4. We find that Agave (*Agave* spp.) was persistently exploited through the last 11,000 years and likely provided an important source of food, alcohol, fiber and building material.
5. We find a shift towards the increasing importance of field crops after ~4,000 BP that is driven by a major increase in the frequency and importance of maize. This observation is consistent with dietary stable isotopes (δ^13^C and δ^15^N) that indicate increasing maize consumption and importance as a staple grain elsewhere in the region [60].
6. Overall, our work is consistent with local experimentation that led to mutualistic and co-evolutionary relationships via direct and indirect selection [21, 66, 67]. This information combined with detailed analysis of domesticated plants from other dry caves in the Americas (e.g. [10–12, 32]) will provide a clearer picture of the complex long-term processes involved with human selection and the domestication process. The chronological framework provided here sets the stage for the finer-grained study of that important question.

## Supporting information

S1 Fig OxCal model, graphical output. (PDF)

S1 File Contains supplementary tables. This file contains a) S1 Table, radiocarbon dates from El Gigante; b) S2 Table, OxCal model results; c) S3 Table, OxCal CQL2 code for model operation; d) S4 Table, stratum thickness measurements from excavation unit profiles (by unit corner); e) S5 Table, calculated stratum volumes (by unit) and depositional rates; f) S6 Table, time series data for proxy records of site utilization; g) S7 Table, correlation matrix for proxy records of site utilization; h) S8 Table, macrofossil counts and depositional rates by occupational phase.
(XLSX)
