# Supplementary material for: Trans–Holocene Bayesian chronology for tree and field crop use from El Gigante rockshelter, Honduras
Source: PLoS One. 2023 Jun 23;18(6):e0287195. doi: 10.1371/journal.pone.0287195 (PMC10289419; doi:10.1371/journal.pone.0287195)

S1 Fig. OxCal model, graphical output.

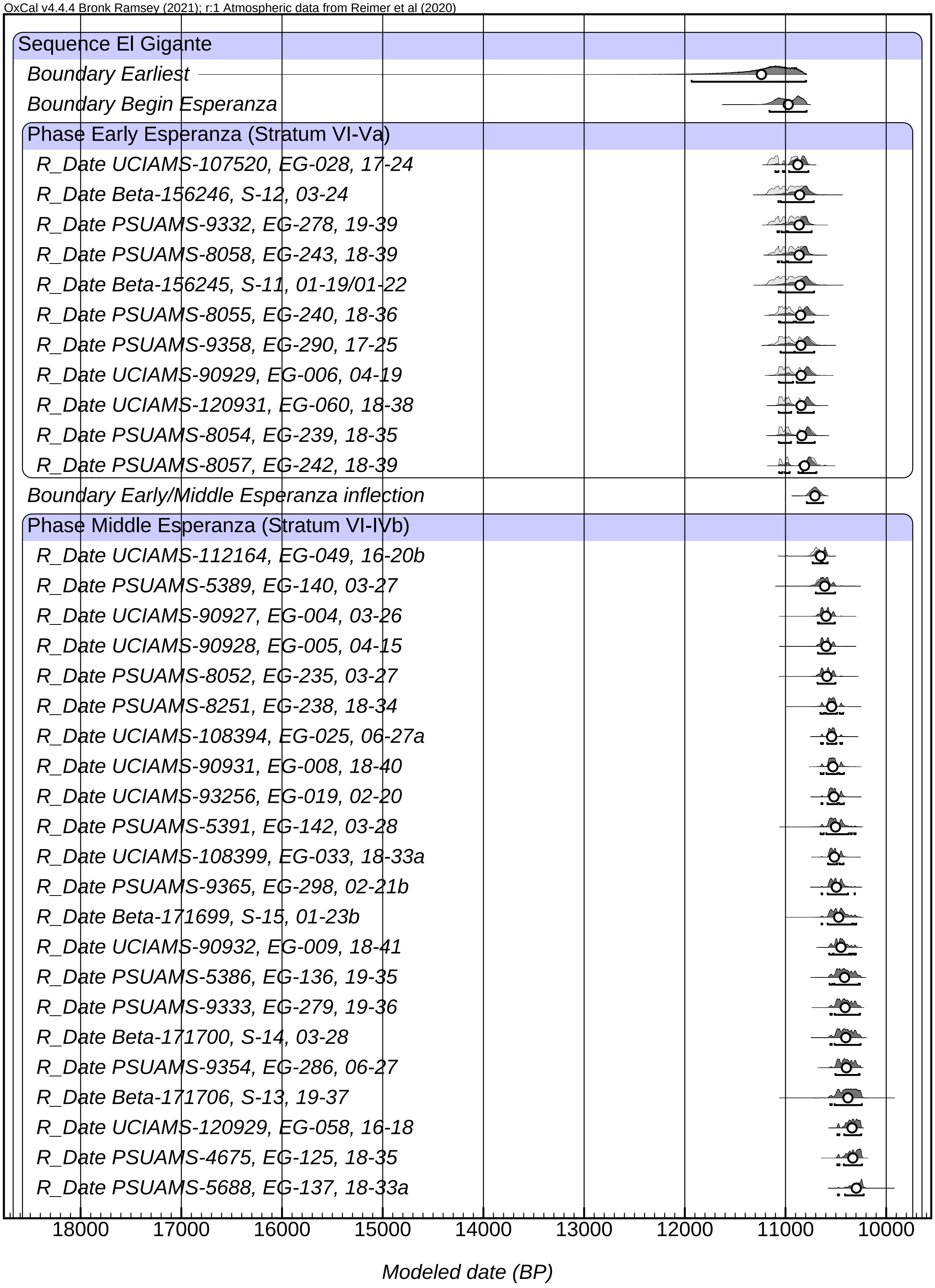

S1 Fig *(continued)*. OxCal model, graphical output.

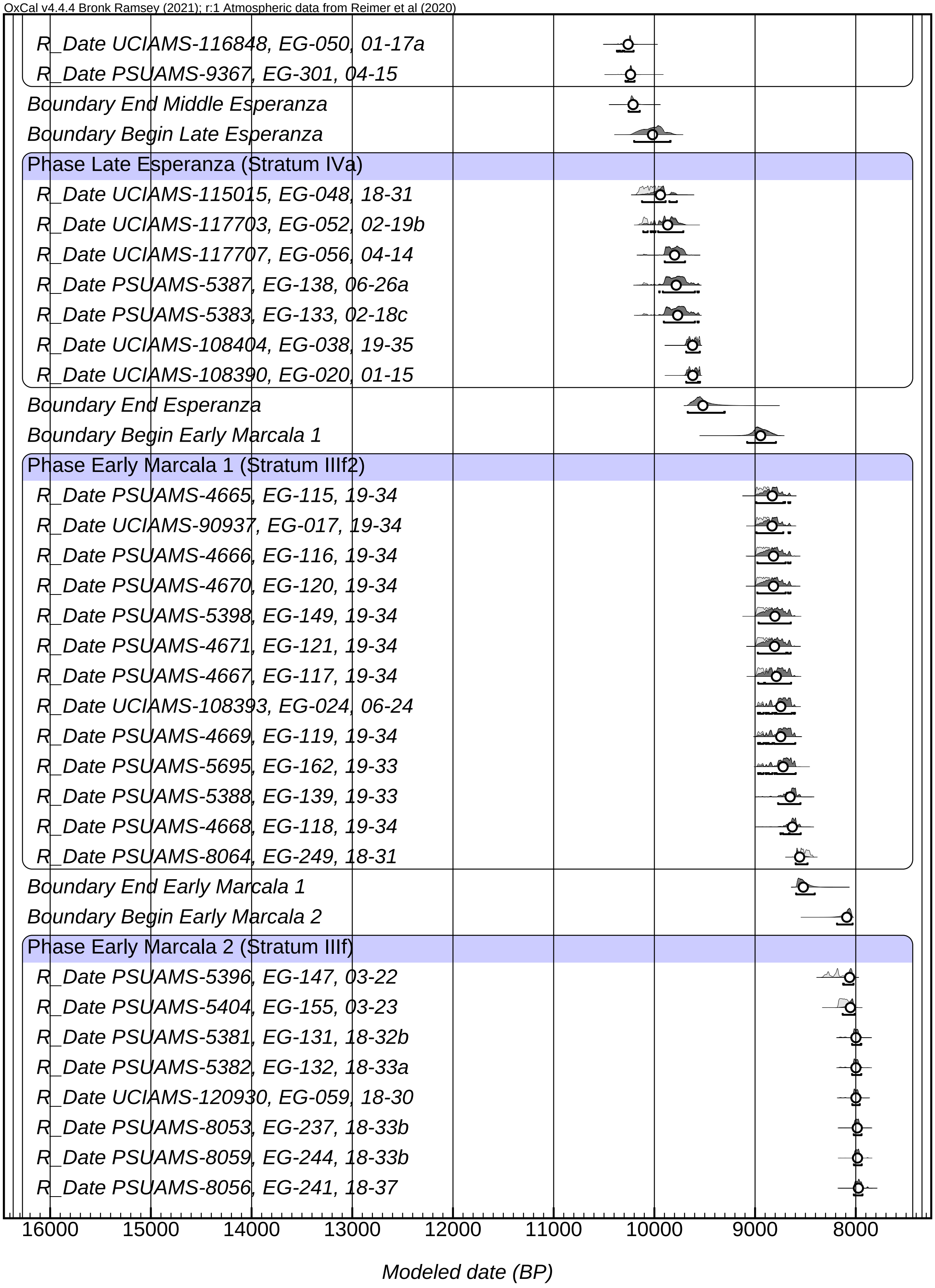

S1 Fig (continued). OxCal model, graphical output.

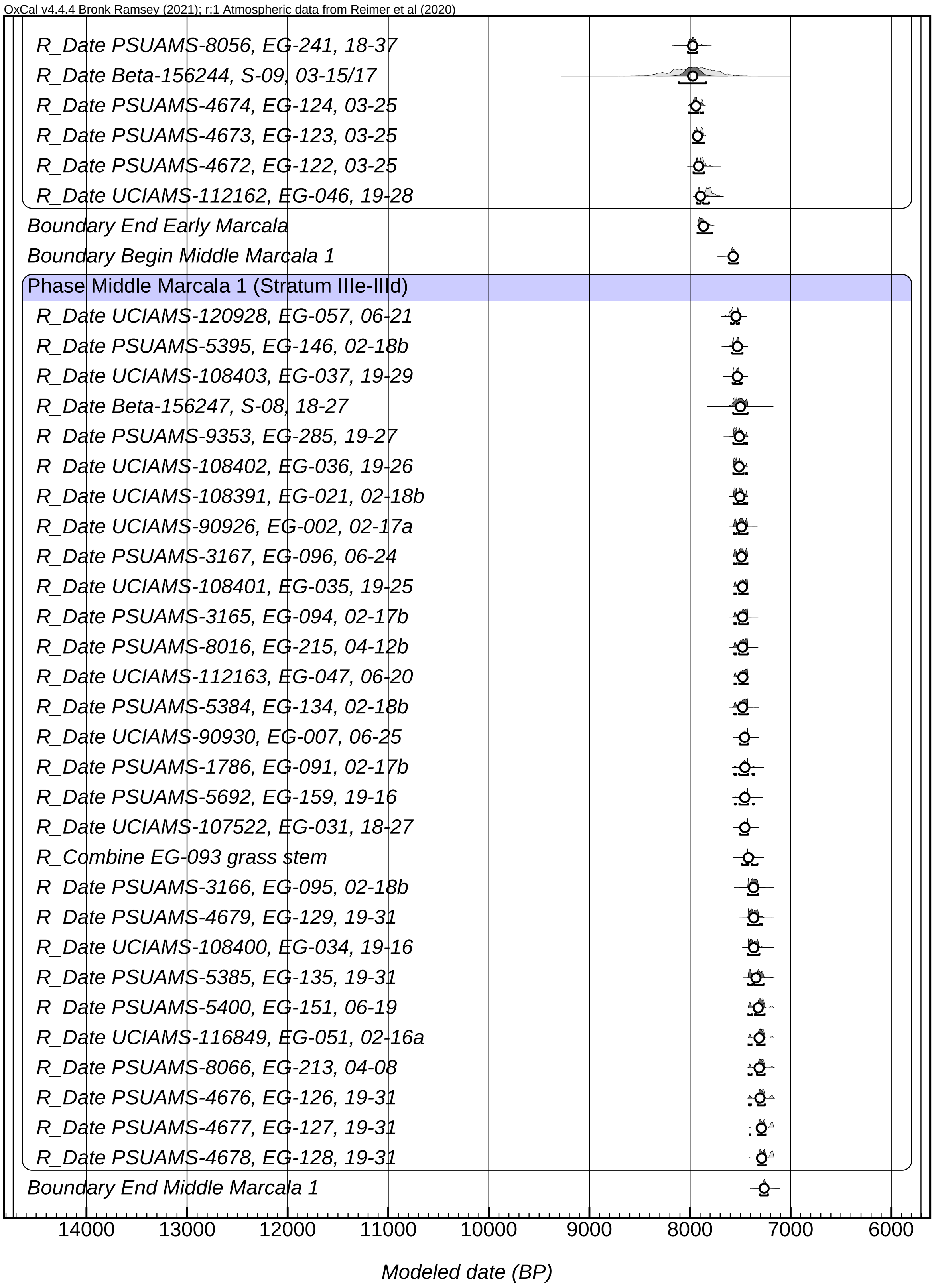

S1 Fig (continued). OxCal model, graphical output.

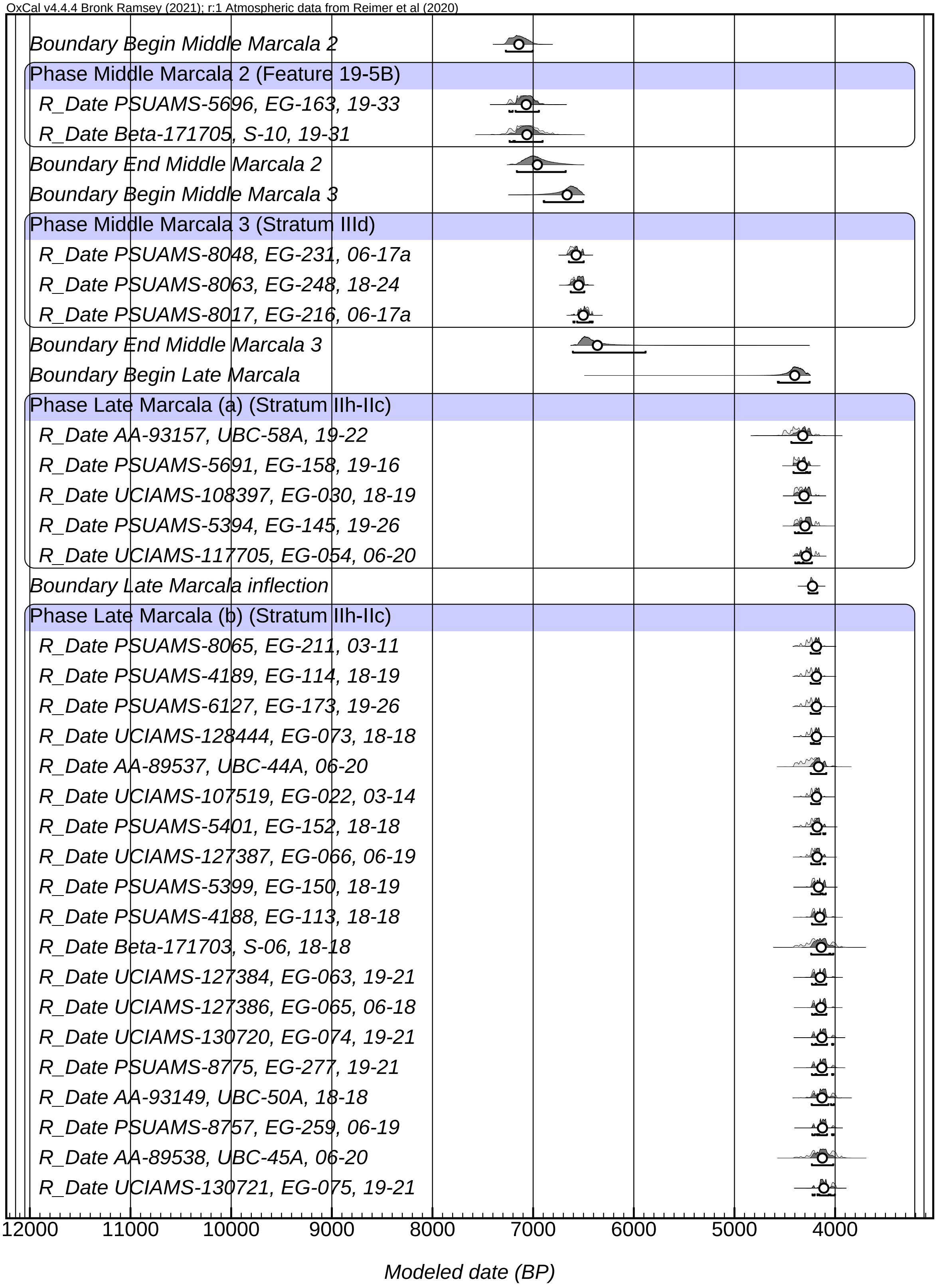

S1 Fig (continued). OxCal model, graphical output.

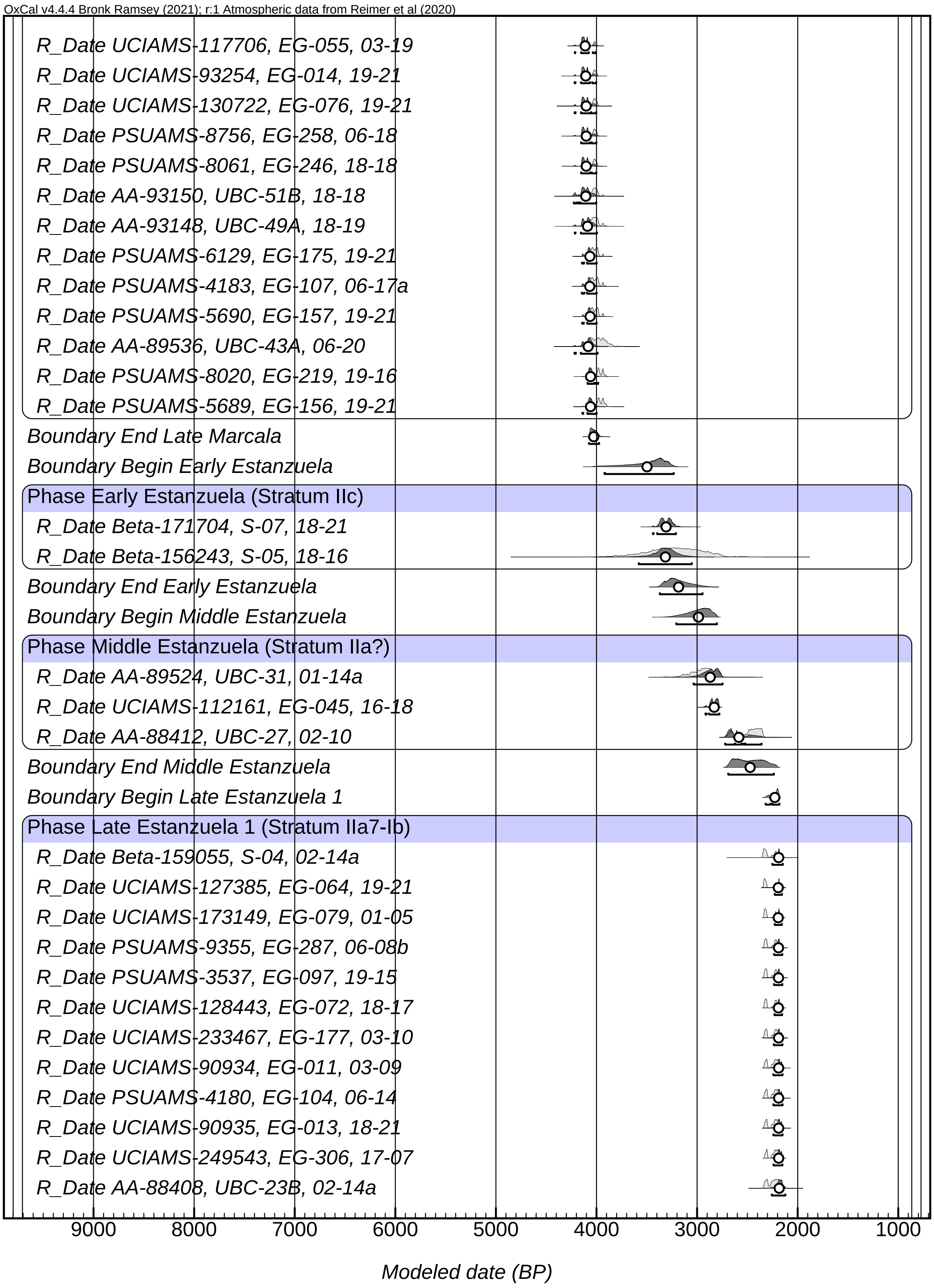

S1 Fig (continued). OxCal model, graphical output.

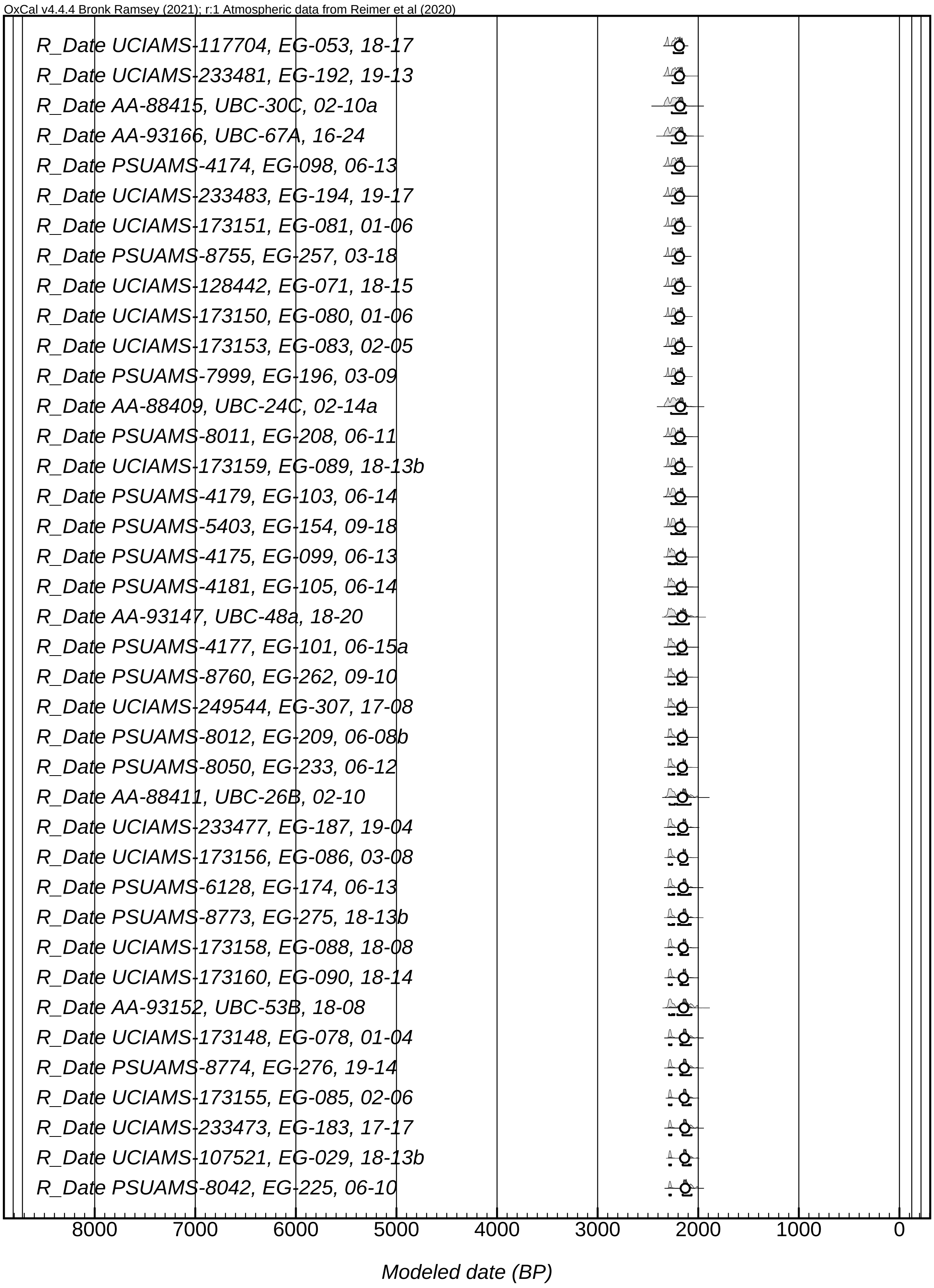

S1 Fig (continued). OxCal model, graphical output.

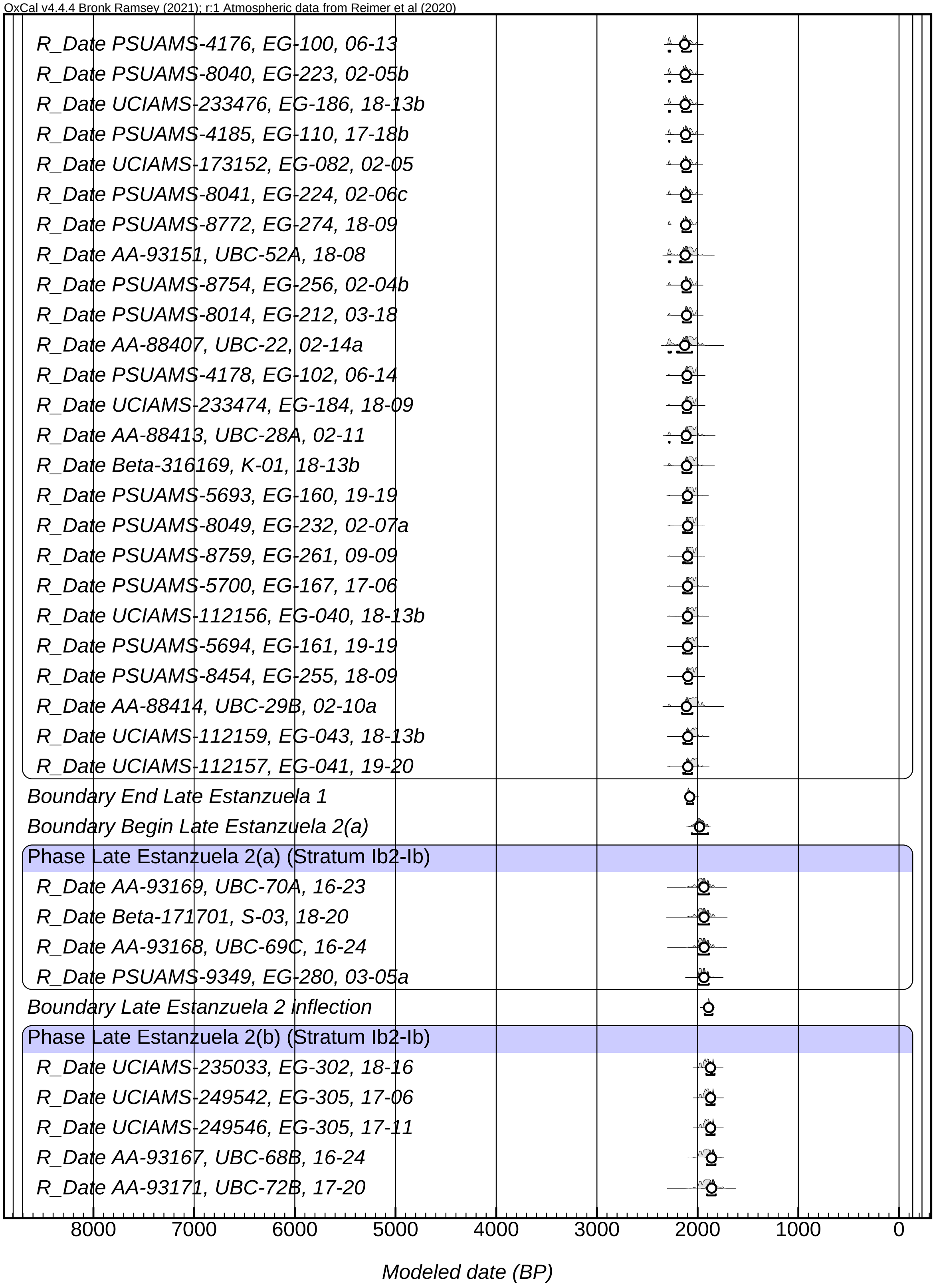

S1 Fig (continued). OxCal model, graphical output.

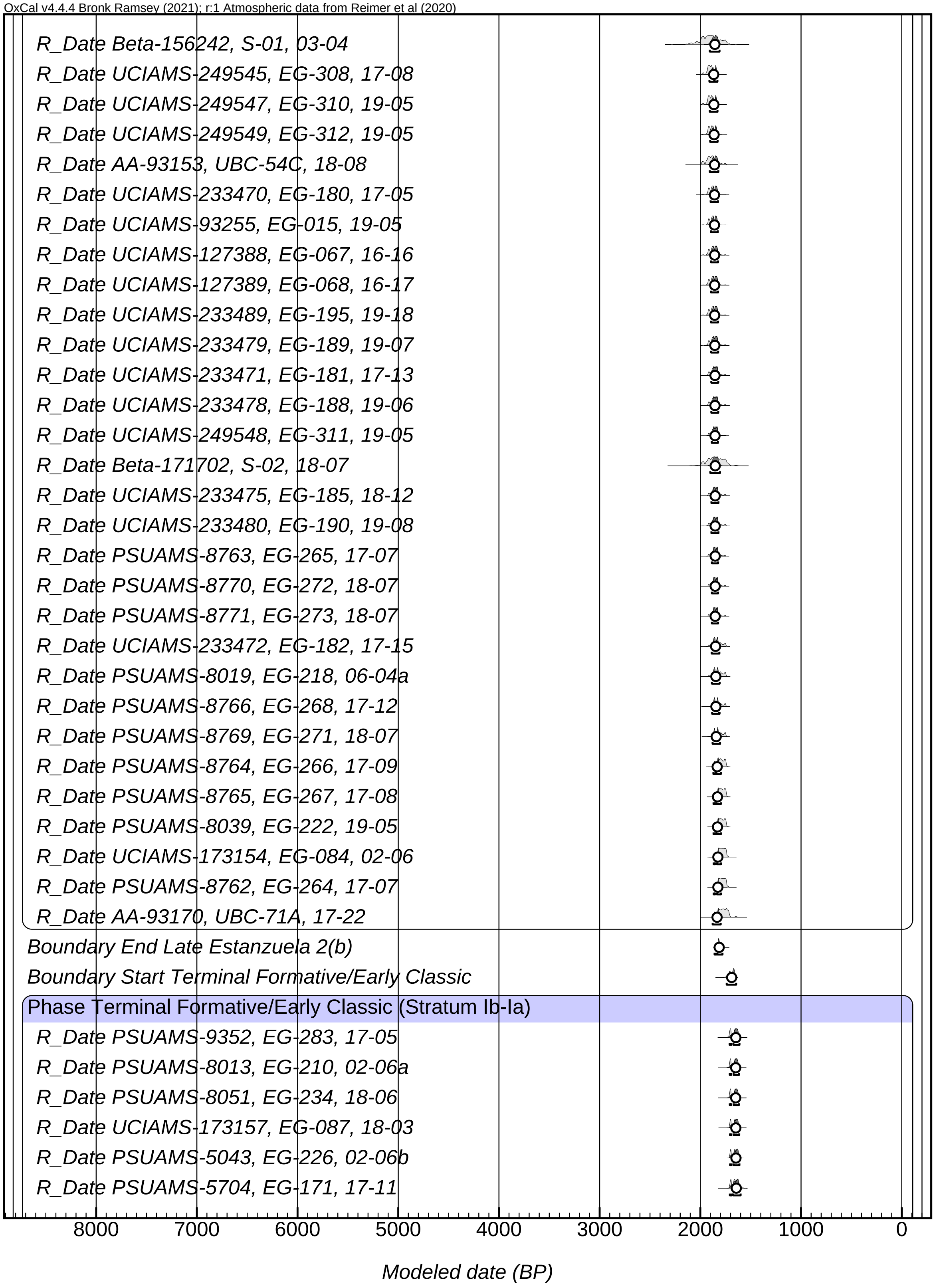

S1 Fig (continued). OxCal model, graphical output.

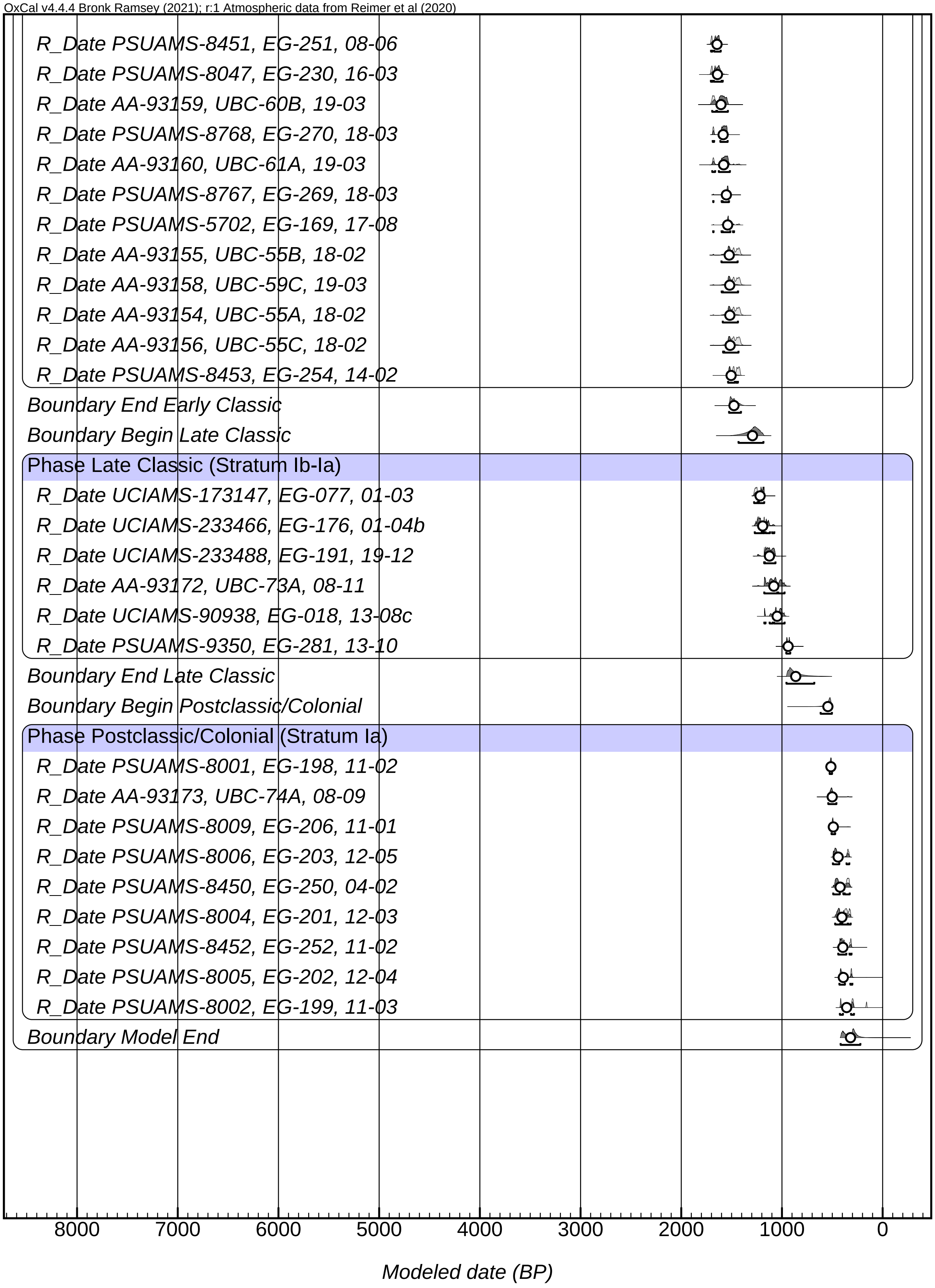

Supplement: S1 Fig — (PDF) [file pone.0287195.s001.pdf]
